# Antidiabetes agents and risk of incident depression in 686,522 people with type 2 diabetes mellitus: A 20-year population-based cohort study

**DOI:** 10.1017/S0033291726104759

**Published:** 2026-06-08

**Authors:** Matthew Tsz Ho Ho, Joe Kwun Nam Chan, Heidi Ka Ying Lo, Catherine Zhiqian Fang, Corine Sau Man Wong, Krystal Chi Kei Lee, Francisco Tsz Tsun Lai, Amy Pui Pui Ng, William Chi Wai Wong, Wing Chung Chang

**Affiliations:** 1 Department of Psychiatry, School of Clinical Medicine, LKS Faculty of Medicine, University of Hong Kong, Hong Kong, China; 2 Department of Psychiatry, United Christian Hospital, Hong Kong, China; 3 Department of Psychiatry, Queen Mary Hospital, Hong Kong, China; 4 School of Public Health, LKS Faculty of Medicine, University of Hong Kong, Hong Kong, China; 5 Centre for Safe Medication Practice and Research, Department of Pharmacology and Pharmacy, LKS Faculty of Medicine, University of Hong Kong, Hong Kong, China; 6 Department of Family Medicine and Primary Care, School of Clinical Medicine, LKS Faculty of Medicine, University of Hong Kong, Hong Kong, China; 7 Advanced Data Analytics for Medical Science (ADAMS) Limited, Hong Kong SAR, China; 8 Department of General Practice, 1 Binhai Road, The University of Hong Kong-Shenzhen Hospital, Shenzhen, China

**Keywords:** type 2 diabetes, depression, anti-hyperglycemic drugs, antidiabetic medications, population-based, electronic health-record

## Abstract

**Background:**

People with diabetes have increased risk of depression that further worsens clinical outcome. However, occurrence of depression among patients with diabetes exposed to antidiabetes agents is under-studied. We investigated whether antidiabetes agents would decrease the risk of depression in people with diabetes.

**Methods:**

This population-based cohort study identified 686,522 patients with incident type-2 diabetes between 2002-2021 in Hong-Kong who were exposed to any antidiabetes agents, using territory-wide medical-record database of public-healthcare services. Associations of exposure to antidiabetes agents and risk of first-diagnosed depression were examined by Cox proportional-hazards models for each individual agent. An array of covariates, including age, sex, calendar-year period, catchment-area, pre-existing physical/psychiatric-comorbidities, average HbA1c-level, diabetic-complications, cardiovascular/lipid-lowering medications, and presence of other antidiabetes agents under investigation was adjusted. Three sets of sensitivity-analyses were conducted by (a) restricting to patients with cumulative drug-exposure ≥90 days and ≥180 days, (b) monotherapy, and (c) incorporating commonly-used non-antidiabetic medications as exposure variables.

**Results:**

Lower risk of new-onset depression was associated with exposure to any antidiabetes agents (HR 0.42, 95%CI 0.39-0.45) compared with no exposure in patients with incident type-2 diabetes. Lower depression risk was associated with exposure to metformin (0.61[0.57-0.66]) compared with no antidiabetes agents, and to pioglitazone (0.25[0.10-0.63]), linagliptin (0.26[0.13-0.52]), and insulin (0.63[0.55-0.72]). Sensitivity-analyses affirmed that lower depression risk was associated with metformin and insulin.

**Conclusion:**

Patients with type-2 diabetes with exposure to several antidiabetes agents is at decreased risk of new-onset depression. Further research is warranted to clarify the depression-reducing mechanisms of antidiabetes agents in this vulnerable population.

## Introduction

Diabetes mellitus affects 537 million people worldwide, with a lifetime risk ranging from 16.3% to 59.6% (Sun et al., 2022). People with type 2 diabetes have ~50% higher risk of developing depression (Lindekilde et al., 2022), resulting in reduced motivation and negative cognitions that are driven by unfavorable lifestyle behaviors, delayed help-seeking, and treatment nonadherence (Berk et al., 2023). The presence of comorbid depression in type 2 diabetes often leads to suboptimal glycemic control, higher complication rates, and increased mortality (Farooqi et al., 2019; Gonzalez et al., 2008; Lustman et al., 2000; Nouwen et al., 2019). Accumulating evidence indicates shared pathophysiological mechanisms underlying both type 2 diabetes and depression, including hypothalamic–pituitary–adrenal axis hyperactivity and immune-inflammatory dysregulation (Moulton, Pickup, & Ismail, 2015). In addition, biological and behavioral factors that are connected with both diabetes and depression, such as obesity and sedentary lifestyles (Tabák, Akbaraly, Batty, & Kivimäki, 2014), could be implicated in poorer clinical outcomes. Notably, evidence suggests that antidiabetes medications might improve depressive symptoms (Moulton, Hopkins, Ismail, & Stahl, 2018) and thereby indirectly reduce their adverse effects on diabetes outcomes (Dragioti et al., 2023).

Antidiabetes agents achieve glycemic control through a range of mechanisms, including amelioration of insulin resistance, stimulation of insulin secretion, and suppression of glucagon secretion (Inzucchi et al., 2012). Many antidiabetes agents, including metformin and thiazolidinediones, may cross the blood–brain barrier (Moulton et al., 2018), and metformin has been found to possess neuroactive activities such as promoting neurotrophins and axonal regeneration in animal models (Houshmand et al., 2019). However, the effect of antidiabetes agents on depression remains understudied. Although some evidence suggests that metformin might exert an antidepressant effect, prior research is hampered by several limitations. First, previous randomized controlled trials were generally of small sample size with restricted inclusion criteria, limiting their generalizability, and study outcomes were mostly defined as depressive symptoms rather than a diagnosis of depressive disorder (Moulton et al., 2018). These depressive symptoms might indicate subclinical depression or diabetes distress (Fisher et al., 2007), which is clinically distinct from depressive disorder. Second, cross-sectional studies did not enroll patients whose depression emerged after the diagnosis of diabetes and thus could not differentiate the temporal sequence between diabetes and new-onset depression (Bojanić et al., 2022; Chin et al., 2020), making it difficult to examine the impact of antidiabetes agents on the risk of incident depression. Third, existing studies were limited by inadequate adjustment of physical comorbidities or the presence of other antidiabetes medications besides the one being studied (Kessing et al., 2020; Wium-Andersen et al., 2022; Yu et al., 2022).

Importantly, even fewer studies have comprehensively investigated a wide spectrum of antidiabetes agents for analysis. Findings from existing studies varied substantially. One study showed that metformin (alone or combined with vildagliptin), glibenclamide, and glimepiride were associated with a reduced risk of depression, while pioglitazone was associated with an increased risk (Kessing et al., 2020). Conversely, another study found that high doses of metformin, sulphonylurea, and insulin were associated with an elevated risk of depression, whereas low doses of metformin, dipeptidyl peptidase-4 (DPP-4) inhibitors, glucagon-like peptide-1 (GLP-1) agonists, and sodium-glucose cotransporter-2 (SGLT-2) inhibitors were associated with decreased risk (Wium-Andersen et al., 2022). Recent meta-analyses have also shown inconsistent results regarding several major medications, including metformin (Zhang et al., 2024), GLP-1 agonists (Cooper et al., 2023), and insulins (Bai, Liu, Li, & Yan, 2018). Taken together, the relationship between antidiabetes agents and incident depression remains to be fully clarified.

To this end, we conducted a population-based cohort study utilizing data retrieved from a territory-wide electronic medical record database of public healthcare services in Hong Kong (HK), a metropolitan city at the southeastern tip of China with a population of ~7.4 million. The study aimed to assess the risk of new-onset depression in patients with incident type 2 diabetes exposed to different antidiabetes agents over a 20-year period. Specifically, we sought to systematically investigate whether exposure to each antidiabetes agent was associated with an altered rate of new-onset depression. A broad array of prespecified confounding variables, including age at incident diabetes, sex, catchment area where patients received medical services, preexisting physical comorbidity, average HbA1c levels over the follow-up period, major diabetic complications, substance and alcohol use disorders, anxiety disorders, obsessive compulsive disorder, and the prescription of antidiabetes agents other than the specified drug under investigation, were adjusted in the analyses.

## Methods

### Data source

We obtained study data from the Clinical Data Analysis and Reporting System (CDARS), a territory-wide electronic health record (EHR) database developed by the Hospital Authority (HA) (Cheung et al., 2007). The HA is a statutory body that manages all public hospitals, and specialist and general outpatient clinics in HK, providing government-subsidized healthcare to all residents, including at least 90% of patients with diabetes in HK. Briefly, CDARS is an integrated EHR system that captures longitudinal clinical data across all HA facilities, including patient demographics, diagnoses, prescriptions, hospital admissions, and outpatient attendances. Patients’ mortality data are retrieved from CDARS through its internal linkage to the regional death registry from the Immigration Department. Each patient is assigned a unique, anonymized identifier by CDARS to facilitate linkage across all medical records while protecting patient privacy. CDARS has been used to generate high-impact population-based studies on various physical diseases and mental disorders (e.g. Chan et al., 2025; Ho et al., 2026), including our previous research on mortality and complication rates, and healthcare utilization associated with preexisting depression and incident diabetes (Ho et al., 2025a, 2025b).

### Study population and patient identification

We identified all individuals aged ≥30 years (to minimize identification of type 1 diabetes, which is associated with a young age at onset of illness) (Jung et al., 2021) who were diagnosed with incident diabetes between 1 January 2002 and 31 December 2021. The ascertainment of diabetes was defined by fulfilling any one of the following criteria: (1) a first-recorded diagnosis of type 2 diabetes by ICD9-CM (code 250, except 250.x1 and 250.x3) for inpatient admission or specialist outpatient attendance, or by the International Classification of Primary Care, Second Edition (code T90), for general outpatient attendance; or (2) prescription of antidiabetic medications, including metformin, sulfonylureas, thiazolidinediones, α-glucosidase inhibitors, meglitinides, and insulin. The onset of diabetes was assigned as the earliest date on which a patient fulfilled the defining criteria during the study period. Patients with records of any diabetes diagnosis (type 1 or type 2) (traced back to 1 January 1998 when the CDARS was implemented) were excluded. To further minimize the inclusion of type 1 diabetes cases, patients with records indicating a diagnosis of type 1 diabetes were also excluded (traced back to 1 January 1998). Individuals with recorded schizophrenia-spectrum disorders (ICD10: F20-F29) or bipolar disorder (F30-F31) as principal diagnoses before or during the study period were also excluded. The study was approved by the Institutional Review Board of the University of Hong Kong/Hospital Authority HK-West Cluster (UW 22–122). Since the data were anonymized and individual patient records were completely unidentifiable during analysis, the requirement for informed consent was waived. The study was reported in accordance with STrengthening the Reporting of OBservational studies in Epidemiology (STROBE) guidelines for cohort studies (Supplementary Table S1).

### Exposure

A total of 39 antidiabetes agents were identified based on medication prescription records of the included diabetic patients. We then classified patients into two groups: the antidiabetes agents-exposed group, consisting of patients who received at least one prescription of any of these agents during the study period, and the antidiabetes agents-unexposed group, comprising patients with no prescriptions for any antidiabetes agents during the same period. The unexposed group served as the comparison for all analyses. Within the exposed group, 10 individual agents, representing the most commonly prescribed antidiabetes regimens in the cohort, were selected for detailed analysis. Patients who were prescribed one of the 39 specified antidiabetes agents during the study period were classified as users of that particular agent. For patients who received more than one of these agents during the study, each of their exposures was analyzed separately in subsequent analyses.

### Follow-up and outcomes

We assessed the occurrence of depressive disorder after ascertainment of incident diabetes. We identified a first-recorded diagnosis of depressive disorder by the ICD10 codes (F32-F33) for psychiatric inpatient admission or outpatient attendance after the ascertainment of diagnosis of incident diabetes from the diabetes cohort. Patients with records of depression before diagnostic assignment of diabetes (i.e. preexisting depressive disorder or dysthymia, F34.1) were excluded. Diabetes patients were followed from the date of diagnostic ascertainment of incident diabetes until there was an occurrence of the first diagnosed depression, 31 December 2021, or the date of death, whichever came first.

### Covariates

Taking into consideration the availability of clinical information adequately captured in the database, an array of preselected candidate covariates was included in the analyses. It comprises patients’ demographic (sex, age at incident diabetes, calendar-year period of ascertainment for incident diabetes, and catchment areas where patients received medical services), and preexisting chronic physical diseases (i.e. physical multimorbidity burden) as quantified by Charlson Comorbidity Index (Charlson, Pompei, Ales, & MacKenzie, 1987; Deyo, Cherkin, & Ciol, 1992), as well as hypertension and dyslipidemia, comorbid substance and alcohol use disorders, anxiety disorders, obsessive compulsive disorder, commonly prescribed non-antidiabetic medications, average HbA1c level over the entire follow-up period, major diabetic complications as classified by the adapted Diabetes Complications Severity Index (DCSI), and the prescription of antidiabetes agents other than the specified drug under investigation. The DCSI is a validated tool in predicting mortality, hospital admissions, and healthcare utilization among patients with diabetes (Young et al., 2008). Seven major DCSI-derived diabetic complications were included, encompassing cardiovascular complications, cerebrovascular complications, peripheral vascular complications, nephropathy, neuropathy, retinopathy, and metabolic complications. The commonly prescribed non-antidiabetic medications at baseline include cardiovascular drugs (aspirin, anticoagulants, digoxin, and antiarrhythmics), antihypertensive drugs (angiotensin-converting enzyme inhibitors, angiotensin receptor blockers, beta-blockers, calcium channel blockers, and diuretics), and lipid-lowering drugs (statins and non-statins). All diabetes complications were identified from both inpatient and outpatient records by ICD9-CM codes (Supplementary Table S2), and were ascertained from the date of diagnostic ascertainment of incident diabetes until the date of death or 31 December 2021, whichever came first. Data for preexisting physical diseases, psychiatric disorders (including anxiety disorders, F40-F41, and obsessive compulsive disorders, F42), and CCI were ascertained from the date of the incident-diabetes diagnosis till 1 January 1998, while data for all of the remaining covariates were ascertained at the time of the incident-diabetes diagnosis. Physical comorbidities and psychiatric comorbidities were identified by ICD9-CM and ICD10 codes, respectively (Supplementary Table S2).

### Statistical analysis

Demographic and baseline characteristics between antidiabetes agents-exposed and antidiabetes agents-unexposed groups were compared using chi-square and independent-samples *t*-tests for categorical and continuous variables, respectively. The association between exposure to antidiabetes agents and the risk of incident depression was analyzed using Cox proportional hazards regression models for exposure to any antidiabetes agents, and separately for each of the 10 commonly prescribed individual agents, with adjustment for confounding variables. Given our large sample size and rich dataset, we chose covariate adjustment to estimate the association because it allows for direct modeling of the hazard. This approach mitigates confounding without the added complexity of propensity-score matching. Three sets of sensitivity analyses were conducted. First, only patients with cumulative exposure to the specified antidiabetes agents of ≥90 and ≥ 180 days were included to ensure an adequate length of exposure to the specified antidiabetes agents. Second, monotherapy analysis was conducted by including patients who had been prescribed the specified antidiabetes agent only within the entire follow-up period. Third, we examined the associations between the prescription of commonly used non-antidiabetic medications and the risk of depression in patients with diabetes. It allows us to evaluate whether the observed associations between antidiabetic agents and depression risk might be attributable to the receipt of healthcare services. The proportional hazards assumptions for all analyses were confirmed using a log-minus-log plot. Results of all Cox proportional hazards regression models were presented as hazard ratios (HRs) in 95% CIs. All statistical analyses were performed using R (version 4.0.2). The threshold of significance for *p*-values was Bonferroni-corrected for multiple comparisons, and *p* < 0.005 was considered statistically significant for the number of drugs involved in the analyses.

## Results

### Characteristics of the study sample

A total of 808,480 patients with incident type 2 diabetes (mean age = 62.9 years, SD = 13.0) were identified during the period from 2002 to 2021 by the medical record database. Among them, 686,522 patients were exposed to any antidiabetes agents (i.e. the antidiabetes agents-exposed group), and 121,958 patients were not exposed to antidiabetes agents (i.e. the antidiabetes agents-unexposed group). Table 1 shows the characteristics of both the antidiabetes agents-exposed and -unexposed groups in the cohort.

**Table 1.** Characteristics of the antidiabetes agents-exposed group and the antidiabetes agents-unexposed group in the incident type 2 diabetes cohort Table 1. long description.

	Antidiabetes agents-exposed group^a^ (*n* = 686,522)	Antidiabetes agents-unexposed group^a^ (*n* = 121,958)	*χ* ^2^/*t*	*P-*value
Demographics				
Age at incident diabetes, years	61.9 (12.7)	68.2 (13.7)	150.9	**<0.001**
Sex			858.2	**<0.001**
Men	365,826 (53.3)	59,444 (48.7)		
Women	320,696 (46.7)	62,514 (51.3)		
Calendar year period of incident diabetes			42,851.2	**<0.001**
2002–2006	185,275 (27.0)	12,233 (10.0)		
2007–2011	157,055 (22.9)	18,540 (15.2)		
2012–2016	168,000 (24.5)	25,638 (21.0)		
2017–2021	176,192 (25.6)	65,557 (53.8)		
Medical comorbidity at baseline				
Hypertension	103,359 (15.1)	46,922 (38.5)	37,530.9	**<0.001**
Dyslipidemia	56,449 (8.2)	29,068 (23.8)	26,685.9	**<0.001**
Charlson comorbidity index (CCI)^b^	2.9 (1.7)	3.9 (2.3)	151.7	**<0.001**
Psychiatric comorbidity at baseline				
Alcohol use disorders	761 (0.1)	230 (0.2)	51.1	**<0.001**
Substance use disorders	977 (0.1)	219 (0.2)	9.7	**0.002**
Anxiety disorders	4,980 (0.7)	1,403 (1.2)	238.8	**<0.001**
Obsessive compulsive disorders	224 (0.0)	51 (0.0)	2.6	0.109
Presence of diabetic complications during follow-up				
Cardiovascular complications	134,594 (19.6)	22,546 (18.5)	82.7	**<0.001**
Cerebrovascular complications	81,905 (11.9)	12,172 (10.0)	383.0	**<0.001**
Peripheral vascular complications	16,541 (2.4)	1,618 (1.3)	552.9	**<0.001**
Nephropathy	78,942 (11.5)	10,488 (8.6)	884.8	**<0.001**
Neuropathy	18,642 (2.7)	1,291 (1.1)	1,179.5	**<0.001**
Retinopathy	38,165 (5.6)	1,287 (1.1)	4,525.9	**<0.001**
Metabolic complications	8,749 (1.3)	300 (0.2)	989.7	**<0.001**
Mean HbA1c level over the entire follow-up	7.05 (1.19)	6.29 (0.69)	265.9	**<0.001**
Medication prescriptions at baseline				
Cardiovascular drugs	147,341 (21.5)	37,207 (30.5)	4,810.7	**<0.001**
Antihypertensive drugs	424,391 (61.8)	91,350 (74.9)	7,677.0	**<0.001**
Lipid-lowering drugs	214,794 (31.3)	49,966 (41.0)	4,408.5	**<0.001**
Catchment areas^c^			1,891.0	**<0.001**
Hong Kong East	65,873 (9.6)	11,406 (9.4)		
Hong Kong West	44,564 (6.5)	6,917 (5.7)		
Kowloon Central	111,678 (16.3)	21,766 (17.8)		
Kowloon East	103,376 (15.1)	22,077 (18.1)		
Kowloon West	131,855 (19.2)	24,093 (19.8)		
New Territories East	119,626 (17.4)	20,873 (17.1)		
New Territories West	109,550 (16.0)	14,826 (12.2)		

*Note*: HbA1c, glycated hemoglobin A1c.

Data are presented in numbers and percentages for all variables, except for age at incident diabetes and Charlson comorbidity index (CCI) score, which are presented in mean (standard deviation).

a Antidiabetes agents-exposed group = patients with incident type 2 diabetes exposed to any antidiabetes agents; antidiabetes agents-unexposed group = patients with incident type 2 diabetes not exposed to any antidiabetes agents.

b Age-adjusted adapted CCI score was computed; diabetes was excluded from CCI score calculations, as it was the disease of interest.

c In Hong Kong, the Hospital Authority delivers public healthcare services for diabetes through inpatient and specialist/general outpatient services organized into seven catchment areas based on geographic locations.

Among the antidiabetes agents-exposed group, six major drug classes were presented, including biguanide, sulphonylurea, thiazolidinedione, DPP-4 inhibitor, SGLT-2 inhibitor, and insulin. In the antidiabetes agents-exposed group, 592,679 subjects were exposed to metformin, 379,258 to gliclazide, 55,621 to glimepiride, 60,854 to pioglitazone, 44,719 to sitagliptin, 43,331 to linagliptin, 28,484 to vildagliptin, 32,168 to empagliflozin, 17,099 to dapagliflozin, and 232,436 to insulin, representing the majority of their respective drug classes.

### Depression risk associated with exposure to antidiabetes agents

Table 2 shows the risk of depression after exposure to each individual agent in patients with incident type 2 diabetes, compared with the antidiabetes agents-unexposed group. Exposure to any antidiabetes agents was associated with a lower risk of depression (HR: 0.42, 95% CI: 0.39–0.45) compared to no antidiabetes agent use in patients with incident diabetes. After Bonferroni correction, a lower risk of depression was observed with exposure to metformin (0.61 [0.57–0.66]), pioglitazone (0.25 [0.10–0.63]), linagliptin (0.26 [0.13–0.52]), and insulin (0.63 [0.55–0.72]), compared with non-use of antidiabetes agents. No significant associations were found between depression risk and other agents.

**Table 2.** Risk of new-onset depression after exposure to antidiabetes agents in patients with incident type 2 diabetes compared with antidiabetes agents-unexposed group Table 2. long description.

Antidiabetes agents	Number of exposed patients	Number of depression events	HR (95% CI)^a^	*P*-value^b^
Any antidiabetes agents	686,522	7,522	0.42 (0.39–0.45)	**<0.001**
Individual agents				
Biguanide				
Metformin	592,679	6,371	0.61 (0.57–0.66)	**<0.001**
Sulphonylurea				
Gliclazide	379,258	4,295	1.12 (1.00–1.24)	0.045
Glimepiride	55,621	383	0.53 (0.30–0.92)	0.026
Thiazolidinedione				
Pioglitazone	60,854	228	0.25 (0.10–0.63)	**0.003**
DPP–4 inhibitor				
Sitagliptin	44,719	317	0.47 (0.23–1.00)	0.051
Linagliptin	43,331	207	0.26 (0.13–0.52)	**<0.001**
Vildagliptin	28,484	147	0.28 (0.11–0.75)	0.011
SGLT–2 inhibitor				
Empagliflozin	32,168	74	0.10 (0.01–0.69)	0.020
Dapagliflozin	17,099	62	0.43 (0.13–1.41)	0.163
Insulin				
Insulin	232,436	1,910	0.63 (0.55–0.72)	**<0.001**

*Note*: 95% CI, 95% confidence interval; DPP-4 inhibitor, dipeptidyl peptidase-4 inhibitors; HR, hazard ratio; SD, standard deviation; SGLT-2 inhibitor, sodium-glucose cotransporter-2 inhibitor.

a Adjustment for age at incident diabetes, gender, calendar year period of diabetes diagnosis, catchment area, Charlson comorbidity index, hypertension, dyslipidemia, cardiovascular complications, cerebrovascular complications, peripheral vascular complications, nephropathy, retinopathy, neuropathy, metabolic complications, average HbA1c level over the entire follow-up period, alcohol and substance dependence, anxiety disorders, obsessive compulsive disorder, cardiovascular drugs, antihypertensive medications, lipid-lowering drugs, and the presence of antidiabetes agents other than the specified drug under investigation.

b The threshold of significance for *p*-values was corrected for multiple comparisons using the Bonferroni method, with *P* < 0.005 (i.e. 0.05/10) considered statistically significant for each antidiabetes agent. Bolded value indicates statistical significance after Bonferroni correction.

### Sensitivity analyses

As shown in Table 3, sensitivity analyses that included patients with cumulative exposure to the specified antidiabetes agent ≥90 days demonstrated a lower depression risk associated with exposure to metformin (0.54 [0.51–0.59]), pioglitazone (0.20 [0.07–0.57]), linagliptin (0.26 [0.12–0.54]), and insulin (0.57 [0.48–0.69]). Similarly, patients with cumulative exposure to the specified antidiabetes agent ≥180 days also showed a reduced depression risk for these drugs (Table 3). In sensitivity analyses restricted to monotherapy, a significant association with lower depression risk was observed for metformin (0.55 [0.51–0.59]) and insulin (0.65 [0.55–0.77]) (Table 4). As shown in Supplementary Table S3, sensitivity analyses examining the association between the prescription of commonly used medications and the risk of incident depression revealed that baseline prescription of lipid-lowering drugs was not significantly associated with risk of incident depression (1.03 [0.97–1.09]). Similarly, cardiovascular drugs showed no significant association (1.05 [0.98–1.12]). However, antihypertensive drugs were associated with a lower risk of incident depression (0.82 [0.78–0.86]).

**Table 3.** Risk of new-onset depression after exposure to antidiabetes agents in patients with incident type 2 diabetes compared with those without exposure to antidiabetes agents in sensitivity analyses with cumulative exposure to the specified antidiabetes agent ≥90 and ≥180 days Table 3. long description.

Antidiabetes agents	Cumulative exposure duration ≥90 days				Cumulative exposure duration ≥180 days			
	Number of exposed patients	Number of depression events	HR (95% CI)^a^	*P*-value^b^	Number of exposed patients	Number of depression events	HR (95% CI)^a^	*P*-value^b^
Biguanide								
Metformin	551,679	5,670	0.54 (0.51–0.59)	**<0.001**	520,006	5,242	0.50 (0.47–0.54)	**<0.001**
Sulphonylurea								
Gliclazide	340,193	3,734	0.99 (0.89–1.12)	0.931	315,839	3,386	0.90 (0.79–1.01)	0.077
Glimepiride	50,721	331	0.45 (0.24–0.86)	0.016	46,171	294	0.40 (0.20–0.82)	0.012
Thiazolidinedione								
Pioglitazone	56,732	197	0.20 (0.07–0.57)	**0.003**	49,227	162	0.17 (0.05–0.58)	**0.004**
DPP–4 inhibitor								
Sitagliptin	39,410	258	0.38 (0.15–0.98)	0.044	34,741	218	0.39 (0.14–1.09)	0.073
Linagliptin	36,761	174	0.26 (0.12–0.54)	**<0.001**	32,331	155	0.24 (0.11–0.52)	**<0.001**
Vildagliptin	25,018	125	0.12 (0.03–0.54)	0.005	21,919	100	0.15 (0.03–0.66)	0.012
SGLT–2 inhibitor								
Empagliflozin	28,781	60	0.10 (0.01–0.75)	0.025	23,999	52	0.13 (0.02–0.95)	0.045
Dapagliflozin	14,756	52	0.16 (0.02–1.17)	0.071	11,843	48	0.19 (0.03–1.44)	0.108
Insulin								
Insulin	122,420	1,150	0.57 (0.48–0.69)	**<0.001**	93,067	845	0.41 (0.32–0.52)	**<0.001**

*Note*: 95% CI, 95% confidence interval; DPP-4 inhibitor, dipeptidyl peptidase-4 inhibitors; HR, hazard ratio; SD, standard deviation; SGLT-2 inhibitor, sodium-glucose cotransporter-2 inhibitor.

a Adjustment for age at incident diabetes, gender, calendar year period of diabetes diagnosis, catchment area, Charlson comorbidity index, hypertension, dyslipidemia, cardiovascular complications, cerebrovascular complications, peripheral vascular complications, nephropathy, retinopathy, neuropathy, metabolic complications, average HbA1c level over the entire follow-up period, alcohol and substance dependence, anxiety disorders, obsessive compulsive disorder, cardiovascular drugs, antihypertensive medications, lipid-lowering drugs, and the presence of antidiabetes agents other than the specified drug under investigation.

b The threshold of significance for *p*-values was corrected for multiple comparisons using the Bonferroni method, with *P* < 0.005 (i.e. 0.05/10) considered statistically significant for each antidiabetes agent. Bolded value indicates statistical significance after Bonferroni correction.

**Table 4.** Risk of new-onset depression after exposure to antidiabetes agents in patients with incident type 2 diabetes compared with those without exposure to antidiabetes agents in the sensitivity analysis of monotherapy Table 4. long description.

Antidiabetes agents	Number of exposed patients	Number of depression events	HR (95% CI)^a^	*P*-value^b^
Biguanide				
Metformin	182,184	2,016	0.55 (0.51–0.59)	**<0.001**
Sulphonylurea				
Gliclazide	20,500	476	1.10 (0.97–1.25)	0.149
Glimepiride	540	7	0.60 (0.19–1.87)	0.376
Thiazolidinedione				
Pioglitazone	231	2	0.61 (0.09–4.35)	0.624
DPP–4 inhibitor				
Sitagliptin	334	4	1.44 (0.46–4.50)	0.530
Linagliptin	–	–	–	–
Vildagliptin	206	2	1.14 (0.28–4.56)	0.858
SGLT–2 inhibitor				
Empagliflozin	–	–	–	–
Dapagliflozin	690	3	1.12 (0.28–4.51)	0.870
Insulin				
Insulin	45,339	323	0.65 (0.55–0.77)	**<0.001**

*Note*: 95% CI, 95% confidence interval; DPP-4 inhibitor, dipeptidyl peptidase-4 inhibitors; HR, hazard ratio; SD, standard deviation; SGLT-2 inhibitor, sodium-glucose cotransporter-2 inhibitor.

a Adjustment for age at incident diabetes, gender, calendar year period of diabetes diagnosis, catchment area, Charlson comorbidity index, hypertension, dyslipidemia, cardiovascular complications, cerebrovascular complications, peripheral vascular complications, nephropathy, retinopathy, neuropathy, metabolic complications, average HbA1c level over the entire follow-up period, alcohol and substance dependence, anxiety disorders, obsessive compulsive disorder, cardiovascular drugs, antihypertensive medications, lipid-lowering drugs, and the presence of antidiabetes agents other than the specified drug under investigation.

b The threshold of significance for *p*-values was corrected for multiple comparisons using the Bonferroni method, with *P* < 0.005 (i.e. 0.05/10) considered statistically significant for each antidiabetes agent. Bolded value indicates statistical significance after Bonferroni correction.

## Discussion

To our knowledge, this investigation is one of the very few population-based cohort studies systematically examining the risk of incident depression associated with a range of commonly prescribed antidiabetes agents among patients with incident type 2 diabetes, using real-world patient data that enhances generalizability of the findings. We demonstrated that exposure to any antidiabetes agents (particularly to metformin and insulin) was significantly associated with a reduced risk of depression in patients with type 2 diabetes compared to those who did not use antidiabetes agents. Remarkably, these findings were estimated after adjusting for baseline physical and psychiatric comorbidities, major diabetic complications, average HbA1c levels, prescription of cardiovascular medications, and the use of antidiabetes agents other than the specified drug under investigation, to eliminate their potential confounding effect on the risk of depression.

Our results aligned with the literature, which indicated a decreased risk of incident depression associated with metformin exposure among patients with diabetes (Kessing et al., 2020; Wium-Andersen et al., 2022; Yu et al., 2022). The differences in the observed magnitude might partly be attributable to methodological differences across studies. In particular, earlier studies were of shorter follow-up duration, smaller sample size, and did not adequately adjust for important confounders including alcohol or substance use disorders (Yu et al., 2022) or the presence of other antidiabetes agents (Kessing et al., 2020; Yu et al., 2022), and some defined the occurrence of depression by prescription of antidepressant medications (Wium-Andersen et al., 2022; Yu et al., 2022), which might be nonspecific to depression diagnosis. Contrary to earlier studies (Kessing et al., 2020; Wium-Andersen et al., 2022), our findings showed a lower risk of depression associated with pioglitazone and insulin, underscoring the importance of confirming the clinical utility of antidiabetes agents in reducing diabetes-depression comorbidity. To date, only two published reports have investigated the occurrence of new-onset depression and its association with a spectrum of antidiabetes agents (Kessing et al., 2020; Wium-Andersen et al., 2022), particularly based on an incident diabetes cohort without preexisting depression to clarify the temporal relationship. Notably, as exposure to antidiabetic agents and increased healthcare contact could lead to higher detection rates of depression, negative associations might be unexpected and warrant careful interpretation. In our case, the observed negative associations suggest that increased healthcare contact does not appear to inflate depression diagnoses among the users of antidiabetic agents, which reduces the concern about collider bias producing protective effects. Importantly, our sensitivity analyses showed that baseline prescription of lipid-lowering and cardiovascular drugs was not significantly associated with a lower risk of depression, suggesting that the reduced depression risk observed with antidiabetic agents is unlikely to be explained simply by the receipt of healthcare services. However, the prescription of antihypertensive drugs was associated with a modestly lower risk of incident depression. This finding should be interpreted with cautions, given the fact that these medications were not the primary exposure of interest. Owing to the limited research on the differential effects of antidiabetes agents in mitigating the risk of depression, our findings of associations between many antidiabetes agents and reduced depression risk underscore the need for further verification and investigation into the mechanisms underlying the potential protective effects of these agents against depression in patients with diabetes.

It is suggested that the potential protective effect of antidiabetes regimens on the development of depression among people with existing diabetes is complex, involving a broad array of mechanistic underpinnings. Antidiabetes agents belonging to insulin-sparing or insulin-sensitizing pharmacological classes have been shown to decrease proinflammatory factors, such as C-reactive proteins and tumor necrosis-alpha factors, thereby reducing oxidative stress associated with depression (Moulton et al., 2018; Scheen, Esser, & Paquot, 2015). Prior studies have also proposed that some antidiabetes agents might exert antidepressant effects through the improvement of cognitive functioning (Scheen et al., 2015). Moreover, accumulating evidence highlights the pleotropic effects of metformin (Dodd et al., 2022), including attenuation of atherosclerosis, reduction of chronic inflammation and oxidative stress, and protection against cerebrovascular diseases, all of which contribute to its neuroprotective benefits. These anti-inflammatory properties of metformin are consistent with the protective effect we observed in the reduction of incident depression associated with metformin exposure. This underscores the need for further research into the anti-inflammatory and neuroprotective effects of antidiabetes agents and their potential clinical utility for depression. More importantly, the COVID-19 pandemic, which overlaps with our study period, has had profound impacts on mental health globally, including increased anxiety, social restrictions, and social isolation. These factors could strain healthcare systems and alter health-seeking behaviors, contributing to the onset of depression. We recognize that the pandemic’s influence constitutes a significant contextual factor that could affect our findings, and further research with more specific data is needed to better understand its impact on depression among patients with diabetes. Nonetheless, our findings suggest that several antidiabetes agents, particularly metformin and insulin, may have a protective effect for people with diabetes who are at risk of developing depression. This could reduce the burden of both conditions by attenuating depressive symptoms and improving patients’ self-management of diabetes (Berk et al., 2023). Alternatively, a previous meta-analysis has demonstrated that effective integrated care for type 2 diabetes can promote sustained improvement in diabetes outcomes by enhancing patient engagement (Lim et al., 2018). This approach may be especially important for diabetes patients with depression, who often face greater management challenges. Therefore, adopting a multilevel management framework is warranted to better address psychiatric symptoms in patients with diabetes.

Several limitations warrant consideration in interpreting the study results. First, it is possible that our study sample might still include some patients with type 1 diabetes, who were ascertained by the presence of recorded prescriptions of antidiabetes agents but without a coded diagnosis from EHR to verify type 2 diabetes (as otherwise these patients would be excluded by case ascertainment via coded diagnosis of type 2 diabetes). Nonetheless, as our study only included patients aged ≥30 years with newly diagnosed diabetes during the study period, and evidence indicates that type 1 diabetes is mainly diagnosed during childhood and adolescence, the rate of misclassification bias by including patients with incident type 1 diabetes in our analysis should be minimized. Although it is possible that a small number of people with type 1 diabetes may have been included, such cases are likely rare and unlikely to substantially affect the overall results. On the other hand, as type 1 and type 2 diabetes have different pathophysiological mechanisms and disease courses, future research is needed to evaluate whether these two types of diabetes may be associated with differential outcomes in terms of the risk of new-onset depression in the context of exposure to antidiabetes agents. Second, data on dosage and combination of antidiabetes agents were not available in our dataset, thereby precluding us from examining depression risk associated with exposure to different dosages or combinations of antidiabetes agents. Future research incorporating dosage-specific data is required to better understand how varying doses of antidiabetes agents may impact depression outcomes in this population.

Third, our definition of depression based on hospital admission or outpatient treatment record captures predominantly more severe cases, and that may introduce a severity bias by excluding milder forms of depression. This focus on a more severe form of depression could underestimate the true incidence of depression in our cohort. It is possible that certain antidiabetes agents might reduce the severity of depressive symptoms without entirely preventing the onset of depression, leading to an underrepresentation of depression cases among the people exposed. Conversely, some agents might have a preventative effect, reducing both the incidence and severity of depression across the spectrum. Future research should incorporate symptom-based assessments to capture a broader range of depression severity. Fourth, an inadequate sample size in some antidiabetes agents, including GLP-1 agonists, meglitinides, and other fixed-dose combinations, precluded us from performing analyses due to small event rates. Given the increasing clinical use of GLP-1 agonists and their potential neuroprotective properties, further investigations with larger sample sizes are warranted to clarify their role in mitigating depression risk among patients with diabetes. Fifth, data on socioeconomic status and lifestyle variables such as physical activity, dietary patterns, and smoking were not adequately recorded in the medical-record database and thus were not included in the analyses. These factors are important determinants of depression and may be associated with both the use of antidiabetes agents and health outcomes. Sixth, similar to most other pharmaco-epidemiological studies, patients’ adherence to prescribed medications was derived from dispensing records, which serve as a proxy for actual medical intake. While dispensing data provides information on prescribed medications, patients’ adherence to the regimens was not captured, and might lead to misclassification of actual medication exposure. This discrepancy may overestimate the actual intake of medications in the cohort and attenuate the observed associations.

Seventh, changes in diabetes treatment recommendations over time might impact prescribing patterns, medication adherence, and clinical outcomes. These temporal variations introduce potential confounding factors that could influence the observed associations between antidiabetic agents and depression. Although we adjusted for calendar year of diagnosis to partially account for these changes, future research should consider explicitly modeling guideline changes to better understand how evolving diabetes treatment practices impact depression risk in people with diabetes. Eighth, data regarding the rate of receipt for guideline-recommended assessments and optimal treatment with antidiabetes agents were not available; hence, the relationship between quality of diabetes care and depression risk could not be evaluated. Future research should incorporate detailed quality of diabetes care data to clarify the relationships among these factors. Ninth, we did not have data regarding corticosteroid use. As corticosteroid use might elevate blood glucose and could confound the relationship between antidiabetic agents and depression outcomes, future research incorporating corticosteroid prescriptions is important to clarify its role in the associations observed. Tenth, as patients who were discontinued from healthcare visits during the follow-up period were censored at their last recorded visit, we recognized that loss to follow-up could introduce bias if it is associated with the likelihood of developing depression. Although sensitivity analyses suggested that our findings remained consistent, the possibility of residual bias due to loss to follow-up cannot be entirely ruled out. Lastly, as our data only encompasses the years 2021 and 2022, the available information is limited for conducting a robust analysis of the specific effects of the COVID-19 pandemic on depression risk within our cohort.

Our findings indicate that people with incident type 2 diabetes who are exposed to several antidiabetes agents are associated with a lower risk of depression compared to those without exposure to antidiabetes agents in a predominantly Chinese population. Our results underscore the potential clinical utility of antidiabetes agents in reducing depression risk among people with diabetes. Since our findings are not consistently affirmed by all sensitivity analyses, more research with larger sample sizes is needed to replicate these results and to clarify the mechanisms by which individual antidiabetes agents may contribute to depression reduction. Further research is also necessary to better understand how various antidiabetes agents influence depression mitigation in this vulnerable population.

## Supporting information

10.1017/S0033291726104759.sm001 Ho et al. supplementary material Ho et al. supplementary material

## Data Availability

The data that support the findings of this study are available from the corresponding author upon reasonable request.

## Supplementary material

The supplementary material for this article can be found at http://doi.org/10.1017/S0033291726104759.

## Table 1. Long description

Starting from the top, the table lists characteristics for antidiabetes agents-exposed group (n equals 686522) and unexposed group (n equals 121958), with test statistics and P-values. Demographics: mean age at incident diabetes is 61.9 years (SD 12.7) for exposed, 68.2 (13.7) for unexposed, test statistic 150.9, P less than 0.001. Sex: men 365826 (53.3 percent) exposed, 59444 (48.7 percent) unexposed; women 320696 (46.7 percent) exposed, 62514 (51.3 percent) unexposed; test statistic 858.2, P less than 0.001. Calendar year periods: 2002–2006, 185275 (27.0 percent) exposed, 12233 (10.0 percent) unexposed; 2007–2011, 157055 (22.9 percent) exposed, 18540 (15.2 percent) unexposed; 2012–2016, 168000 (24.5 percent) exposed, 25638 (21.0 percent) unexposed; 2017–2021, 176192 (25.6 percent) exposed, 65557 (53.8 percent) unexposed; test statistic 42851.2, P less than 0.001. Medical comorbidity at baseline: hypertension 103359 (15.1 percent) exposed, 46922 (38.5 percent) unexposed, test statistic 37530.9, P less than 0.001; dyslipidemia 56449 (8.2 percent) exposed, 29068 (23.8 percent) unexposed, test statistic 26685.9, P less than 0.001; Charlson comorbidity index mean 2.9 (1.7) exposed, 3.9 (2.3) unexposed, test statistic 151.7, P less than 0.001. Psychiatric comorbidity: alcohol use disorders 761 (0.1 percent) exposed, 230 (0.2 percent) unexposed, test statistic 51.1, P less than 0.001; substance use disorders 977 (0.1 percent) exposed, 219 (0.2 percent) unexposed, test statistic 9.7, P equals 0.002; anxiety disorders 4980 (0.7 percent) exposed, 1403 (1.2 percent) unexposed, test statistic 238.8, P less than 0.001; obsessive compulsive disorders 224 (0.0 percent) exposed, 51 (0.0 percent) unexposed, test statistic 2.6, P equals 0.109. Diabetic complications during follow-up: cardiovascular 134594 (19.6 percent) exposed, 22546 (18.5 percent) unexposed, test statistic 82.7, P less than 0.001; cerebrovascular 81905 (11.9 percent) exposed, 12172 (10.0 percent) unexposed, test statistic 383.0, P less than 0.001; peripheral vascular 16541 (2.4 percent) exposed, 1618 (1.3 percent) unexposed, test statistic 552.9, P less than 0.001; nephropathy 78942 (11.5 percent) exposed, 10488 (8.6 percent) unexposed, test statistic 884.8, P less than 0.001; neuropathy 18642 (2.7 percent) exposed, 1291 (1.1 percent) unexposed, test statistic 1179.5, P less than 0.001; retinopathy 38165 (5.6 percent) exposed, 1287 (1.1 percent) unexposed, test statistic 4525.9, P less than 0.001; metabolic complications 8749 (1.3 percent) exposed, 300 (0.2 percent) unexposed, test statistic 989.7, P less than 0.001. Mean H b A 1 c over follow-up: 7.05 (1.19) exposed, 6.29 (0.69) unexposed, test statistic 265.9, P less than 0.001. Medication prescriptions at baseline: cardiovascular drugs 147341 (21.5 percent) exposed, 37207 (30.5 percent) unexposed, test statistic 4810.7, P less than 0.001; antihypertensive drugs 424391 (61.8 percent) exposed, 91350 (74.9 percent) unexposed, test statistic 7677.0, P less than 0.001; lipid-lowering drugs 214794 (31.3 percent) exposed, 49966 (41.0 percent) unexposed, test statistic 4408.5, P less than 0.001. Catchment areas: Hong Kong East 65873 (9.6 percent) exposed, 11406 (9.4 percent) unexposed; Hong Kong West 44564 (6.5 percent) exposed, 6917 (5.7 percent) unexposed; Kowloon Central 111678 (16.3 percent) exposed, 21766 (17.8 percent) unexposed; Kowloon East 103376 (15.1 percent) exposed, 22077 (18.1 percent) unexposed; Kowloon West 131855 (19.2 percent) exposed, 24093 (19.8 percent) unexposed; New Territories East 119626 (17.4 percent) exposed, 20873 (17.1 percent) unexposed; New Territories West 109550 (16.0 percent) exposed, 14826 (12.2 percent) unexposed. All P-values less than 0.001 unless otherwise specified. Data are numbers and percentages, except age and C C I, which are mean and standard deviation.

Navigate back to Table 1..

## Table 2. Long description

The table lists antidiabetes agents in the first column, followed by number of exposed patients, number of depression events, hazard ratio with 95 percent confidence interval, and p-value. For any antidiabetes agents, 686,522 patients were exposed, with 7,522 depression events, hazard ratio 0.42 (0.39 to 0.45), p-value less than 0.001. For metformin, 592,679 exposed, 6,371 events, hazard ratio 0.61 (0.57 to 0.66), p-value less than 0.001. For gliclazide, 379,258 exposed, 4,295 events, hazard ratio 1.12 (1.00 to 1.24), p-value 0.045. For glimepiride, 55,621 exposed, 383 events, hazard ratio 0.53 (0.30 to 0.92), p-value 0.026. For pioglitazone, 60,854 exposed, 228 events, hazard ratio 0.25 (0.10 to 0.63), p-value 0.003. For sitagliptin, 44,719 exposed, 317 events, hazard ratio 0.47 (0.23 to 1.00), p-value 0.051. For linagliptin, 43,331 exposed, 207 events, hazard ratio 0.26 (0.13 to 0.52), p-value less than 0.001. For vildagliptin, 28,484 exposed, 147 events, hazard ratio 0.28 (0.11 to 0.75), p-value 0.011. For empagliflozin, 32,168 exposed, 74 events, hazard ratio 0.10 (0.01 to 0.69), p-value 0.020. For dapagliflozin, 17,099 exposed, 62 events, hazard ratio 0.43 (0.13 to 1.41), p-value 0.163. For insulin, 232,436 exposed, 1,910 events, hazard ratio 0.63 (0.55 to 0.72), p-value less than 0.001. Statistically significant results after Bonferroni correction are bolded. Adjustment factors include age, gender, year of diagnosis, comorbidities, complications, average H b A 1 c, substance dependence, psychiatric disorders, and concurrent medications.

Navigate back to Table 2..

## Table 3. Long description

The table lists antidiabetes agents in rows, grouped by drug class: Biguanide (Metformin), Sulphonylurea (Gliclazide, Glimepiride), Thiazolidinedione (Pioglitazone), D P P dash 4 inhibitor (Sitagliptin, Linagliptin, Vildagliptin), S G L T dash 2 inhibitor (Empagliflozin, Dapagliflozin), and Insulin. For each agent, data are presented for cumulative exposure duration of at least 90 days and at least 180 days. Columns for each duration include number of exposed patients, number of depression events, hazard ratio with 95 percent confidence interval, and p-value. Metformin shows 551,679 patients exposed for at least 90 days with 5,670 depression events, hazard ratio 0.54 (0.51 to 0.59), p-value less than 0.001; for at least 180 days, 520,006 patients, 5,242 events, hazard ratio 0.50 (0.47 to 0.54), p-value less than 0.001. Gliclazide has 340,193 patients, 3,734 events, hazard ratio 0.99 (0.89 to 1.12), p-value 0.931 for 90 days; 315,839 patients, 3,386 events, hazard ratio 0.90 (0.79 to 1.01), p-value 0.077 for 180 days. Glimepiride shows 50,721 patients, 331 events, hazard ratio 0.45 (0.24 to 0.86), p-value 0.016 for 90 days; 46,171 patients, 294 events, hazard ratio 0.40 (0.20 to 0.82), p-value 0.012 for 180 days. Pioglitazone has 56,732 patients, 197 events, hazard ratio 0.20 (0.07 to 0.57), p-value 0.003 for 90 days; 49,227 patients, 162 events, hazard ratio 0.17 (0.05 to 0.58), p-value 0.004 for 180 days. Sitagliptin shows 39,410 patients, 258 events, hazard ratio 0.38 (0.15 to 0.98), p-value 0.044 for 90 days; 34,741 patients, 218 events, hazard ratio 0.39 (0.14 to 1.09), p-value 0.073 for 180 days. Linagliptin has 36,761 patients, 174 events, hazard ratio 0.26 (0.12 to 0.54), p-value less than 0.001 for 90 days; 32,331 patients, 155 events, hazard ratio 0.24 (0.11 to 0.52), p-value less than 0.001 for 180 days. Vildagliptin shows 25,018 patients, 125 events, hazard ratio 0.12 (0.03 to 0.54), p-value 0.005 for 90 days; 21,919 patients, 100 events, hazard ratio 0.15 (0.03 to 0.66), p-value 0.012 for 180 days. Empagliflozin has 28,781 patients, 60 events, hazard ratio 0.10 (0.01 to 0.75), p-value 0.025 for 90 days; 23,999 patients, 52 events, hazard ratio 0.13 (0.02 to 0.95), p-value 0.045 for 180 days. Dapagliflozin shows 14,756 patients, 52 events, hazard ratio 0.16 (0.02 to 1.17), p-value 0.071 for 90 days; 11,843 patients, 48 events, hazard ratio 0.19 (0.03 to 1.44), p-value 0.108 for 180 days. Insulin has 122,420 patients, 1,150 events, hazard ratio 0.57 (0.48 to 0.69), p-value less than 0.001 for 90 days; 93,067 patients, 845 events, hazard ratio 0.41 (0.32 to 0.52), p-value less than 0.001 for 180 days. Statistically significant p-values after Bonferroni correction are bolded. Hazard ratios below 1 indicate reduced risk of new-onset depression compared to patients not exposed to the agent.

Navigate back to Table 3..

## Table 4. Long description

From the top, the table is divided by antidiabetes agent classes: Biguanide, Sulphonylurea, Thiazolidinedione, D P P dash 4 inhibitor, S G L T dash 2 inhibitor, and Insulin. Under Biguanide, Metformin shows 182,184 exposed patients, 2,016 depression events, hazard ratio 0.55 (0.51–0.59), p-value less than 0.001, indicating statistically significant reduction in depression risk. Sulphonylurea includes Gliclazide with 20,500 exposed, 476 events, hazard ratio 1.10 (0.97–1.25), p-value 0.149; Glimepiride with 540 exposed, 7 events, hazard ratio 0.60 (0.19–1.87), p-value 0.376. Thiazolidinedione lists Pioglitazone with 231 exposed, 2 events, hazard ratio 0.61 (0.09–4.35), p-value 0.624. D P P dash 4 inhibitor includes Sitagliptin with 334 exposed, 4 events, hazard ratio 1.44 (0.46–4.50), p-value 0.530; Linagliptin with missing data; Vildagliptin with 206 exposed, 2 events, hazard ratio 1.14 (0.28–4.56), p-value 0.858. S G L T dash 2 inhibitor lists Empagliflozin with missing data; Dapagliflozin with 690 exposed, 3 events, hazard ratio 1.12 (0.28–4.51), p-value 0.870. Insulin shows 45,339 exposed, 323 events, hazard ratio 0.65 (0.55–0.77), p-value less than 0.001, also statistically significant. Statistically significant hazard ratios after Bonferroni correction are bolded. Missing values are indicated by dashes.

Navigate back to Table 4..
