# Supplementary material for: Antidiabetes agents and risk of incident depression in 686,522 people with type 2 diabetes mellitus: A 20-year population-based cohort study
Source: Psychol Med. 2026 Jun 8;56:e183. doi: 10.1017/S0033291726104759 (PMC13247788; doi:10.1017/S0033291726104759)
Supplement: Ho et al. supplementary material [file S0033291726104759sup001.docx]

**Supplementary Materials**

**Table S1.** The STROBE checklist.

**Table S2.** Operational definitions according to ICD9-CM or ICD10 codes for diagnoses.

**Table S3.** Risk of new-onset depression after exposure to commonly-prescribed non-antidiabetic medications at baseline for patients with incident type 2 diabetes compared with those who are unexposed.

**Table S1.**

The STROBE statements.

|  | **Item No.** | **STROBE items** | **Location in manuscript where items are reported** |
| --- | --- | --- | --- |
| **Title and abstract** | | | |
|  | 1 | (a) Indicate the study’s design with a commonly used term in the title or the abstract (b) Provide in the abstract an informative and balanced summary of what was done and what was found | Abstract |
| **Introduction** | | | |
| Background/ rationale | 2 | Explain the scientific background and rationale for the investigation being reported | Introduction, 1^st^ to 3^rd^ paragraphs |
| Objectives | 3 | State specific objectives, including any prespecified hypotheses | Introduction, 4^th^ paragraph |
| **Methods** | | | |
| Study design | 4 | Present key elements of study design early in the paper | Methods section/ data sources and study design |
| Setting | 5 | Describe the setting, locations, and relevant dates, including periods of recruitment, exposure, follow-up, and data collection | Methods section/ data sources and study design |
| Participants | 6 | *(a)* Give the eligibility criteria, and the sources and methods of selection of participants. Describe methods of follow-up  *(b)* For matched studies, give matching criteria and number of exposed and unexposed | Methods section/ data sources and study design |
| Variables | 7 | Clearly define all outcomes, exposures, predictors, potential confounders, and effect modifiers. Give diagnostic criteria, if applicable. | Methods section/ exposure, follow-up and outcomes |
| Data sources/ measurement | 8 | For each variable of interest, give sources of data and details of methods of assessment (measurement).  Describe comparability of assessment methods if there is more than one group | Methods section/ data sources and study design |
| Bias | 9 | Describe any efforts to address potential sources of bias | Methods section/ statistical analysis |
| Study size | 10 | Explain how the study size was arrived at | Methods section/ study population |
| Quantitative variables | 11 | Explain how quantitative variables were handled in the analyses. If applicable, describe which groupings were chosen, and why | Methods section/ statistical analysis |
| Statistical methods | 12 | (a) Describe all statistical methods, including those used to control for confounding  (b) Describe any methods used to examine subgroups and interactions  (c) Explain how missing data were addressed  (d) If applicable, explain how loss to follow-up was addressed  (e) Describe any sensitivity analyses | Methods section/ statistical analysis |
| **Results** | | | |
| Participants | 13 | (a) Report the numbers of individuals at each stage of the study (*e.g.*, numbers potentially eligible, examined for eligibility, confirmed eligible, included in the study, completing follow-up, and analyzed)  (b) Give reasons for non-participation at each stage.  (c) Consider use of a flow diagram | Results, 1^st^ paragraph |
| Descriptive data | 14 | (a) Give characteristics of study participants (*e.g.*, demographic, clinical, social) and information on exposures and potential confounders  (b) Indicate the number of participants with missing data for each variable of interest  (c) Summarize follow-up time (*e.g.*, average and total amount) | Results, 1^st^ paragraph, Table 1 |
| Outcome data | 15 | *Cohort study* - Report numbers of outcome events or summary measures over time | Results, 2^nd^ paragraph, Table 2 |
| Main results | 16 | (a) Give unadjusted estimates and, if applicable, confounder-adjusted estimates and their precision (e.g., 95% confidence interval). Make clear which confounders were adjusted for and why they were included  (b) Report category boundaries when continuous variables were categorized  (c) If relevant, consider translating estimates of relative risk into absolute risk for a meaningful time period | Results, 2^nd^ paragraph, Table 2 |
| Other analyses | 17 | Report other analyses done—e.g., analyses of subgroups and interactions, and sensitivity analyses | Results, 3^rd^ paragraph, Table 3-4, supplementary table S3 |
| **Discussion** | | | |
| Key results | 18 | Summarize key results with reference to study objectives | Discussion, 1^st^ paragraph |
| Limitations | 19 | Discuss limitations of the study, taking into account sources of potential bias or imprecision. Discuss both direction and magnitude of any potential bias | Discussion, 4^th^ to 6^th^ paragraphs |
| Interpretation | 20 | Give a cautious overall interpretation of results considering objectives, limitations, multiplicity of analyses, results from similar studies, and other relevant evidence | Discussion, 2^nd^ paragraph |
| Generalizability | 21 | Discuss the generalizability (external validity) of the study results | Discussion, 3^rd^ paragraph |
| **Other Information** | | | |
| Funding | 22 | Give the source of funding and the role of the funders for the present study and, if applicable, for the original study on which the present article is based | Role of the funding source |

**Note:** An Explanation and Elaboration article discusses each checklist item and gives methodological background and published examples of transparent reporting. The STROBE checklist is best used in conjunction with this article (freely available on the Web sites of PLoS Medicine at http://www.plosmedicine.org/, Annals of Internal Medicine at http://www.annals.org/, and Epidemiology at http://www.epidem.com/). Information on the STROBE Initiative is available at http://www.strobe-statement.org.

**Table S2.**

Operational definitions according to ICD9-CM or ICD10 codes for diagnoses.

| Diagnoses | Operational definitions |
| --- | --- |
| **Psychiatric disorders** | **ICD10 codes** ^a^ |
| Schizophrenia-spectrum disorder | F20–F29 |
| Bipolar disorder | F30–F31 |
| Depressive disorder (i.e., major depressive disorder) | F32–F33 |
| Dysthymia | F34.1 |
| Substance use disorder | F11–F19 |
| Alcohol use disorder | F10 |
| Anxiety disorders | F40–F41 |
| Obsessive-compulsive disorder | F42 |
| **Physical diseases** | **ICD9-CM codes** ^a^ |
| Diabetes mellitus | 250, except 250.x1 and 250.x3 |
| Diabetes complications adapted from Diabetes Complication Severity Index (DCSI) | |
| Cardiovascular complications | 410, 411, 413, 414, 427.1, 427.3–427.5, 428, 429.2, 440, 440.23, 440.24, 441 |
| Cerebrovascular complications | 431, 433, 434, 435, 436 |
| Peripheral vascular complications | 0.40, 250.7, 442.3, 443.81, 443.9, 444.22, 707.1, 785.4, 892.1 |
| Retinopathy | 250.5, 361, 362.01, 362.02, 362.1, 362.53, 362.81, 362.82, 362.83, 369, 379.23 |
| Nephropathy | 250.4, 580, 581, 581.81, 582, 583, 585, 586, 593.9 |
| Neuropathy | 250.6, 337.0, 337.1, 354.0 – 355.9, 356.9, 357.2, 358.1, 458.0, 536.3, 564.5, 596.54, 713.5, 951.0, 951.1, 951.3 |
| Metabolic complications | 250.1, 250.2, 250.3 |
| Physical comorbidities adapted from Charlson Comorbidity Index | |
| Myocardial infarction | 410–410.9, 412 |
| Congestive heart failure | 428–428.9 |
| Peripheral vascular disease | 441, 443.9, 785.4, V43.4 |
| Cerebrovascular disease | 430–438 |
| Dementia | 290 |
| Chronic pulmonary disease | 490–496, 500–505, 506.4 |
| Rheumatological disease | 710.0, 710.1, 710.4, 714.0–714.2, 714.8, 725 |
| Peptic ulcer disease | 531–534 |
| Mild liver disease | 571.2, 571.4, 571.5-571.6 |
| Hemiplegia or paraplegia | 322, 344.1 |
| Renal disease | 582-583, 585–586, 588 |
| Any malignancy including leukemia or lymphoma | 140–172, 174–195, 200–208 |
| Moderate or severe liver disease | 572.2–572.8 |
| Metastatic cancers | 196-198, 199.0–199.1 |
| Hypertension | 401, 405 |
| Dyslipidemia | 272 |

^a^ In Clinical Data Analysis and Reporting System (CDARS), physical diseases are categorized by ICD9-CM codes and psychiatric disorders are categorized by

ICD10 codes.

**Table S3.**

Risk of new-onset depression after exposure to commonly-prescribed non-antidiabetic medications at baseline for patients with

incident type 2 diabetes compared with those who are unexposed.

| Exposure to commonly-prescribed non-antidiabetic medications at baseline | Number of exposed patients | Number of depression events | HR (95% CI) ^1^ | *P* value ^2^ |
| --- | --- | --- | --- | --- |
| Lipid-lowering drugs | 264,760 | 2,263 | 1.03 (0.97-1.09) | 0.372 |
| Anti-hypertensive drugs | 515,741 | 5,088 | 0.82 (0.78-0.86) | **<0.001** |
| Cardiovascular drugs | 184,548 | 1,719 | 1.05 (0.98-1.12) | 0.135 |

95% CI = 95% confidence interval; HR = hazard ratio; SD = standard deviation

^1^ adjustment for age at incident diabetes, gender, calendar year period of diabetes diagnosis, catchment area, Charlson

comorbidity index, hypertension, dyslipidemia, cardiovascular complications, cerebrovascular complications, peripheral

vascular complications, nephropathy, retinopathy, neuropathy, metabolic complications, average HbA1c levels over the

entire follow-up period, alcohol and substance dependence, anxiety disorders, and obsessive compulsive disorder.

^2^ the threshold of significance for *p* values was corrected for multiple comparisons using the Bonferroni method, with *P*<0.017

(i.e., 0.05/3) considered statistically significant for each antidiabetes agent. Bolded value indicates statistical significance after

Bonferroni correction.
